# Subdural contrast extravasation after percutaneous coronary intervention mimicking acute subdural hematoma: A case report

**DOI:** 10.1097/MD.0000000000025583

**Published:** 2021-04-30

**Authors:** Jinghua Chen, Ping Xie, Jian Huang, Eryan Sheng, Kefu Liu

**Affiliations:** a Department of Radiology, Taicang City Hospital of Traditional Chinese Medicine, Taicang; b Department of Radiology, The Affiliated Suzhou Hospital of Nanjing Medical University, Suzhou, Jiangsu, China.

**Keywords:** acute subdural hematoma, case report, computed tomography, subarachnoid hemorrhage, subdural contrast extravasation

## Abstract

**Rationale:**

Subdural contrast extravasation (SCE) is a rare and possible complication following the intravascular injection of a contrast agent. We report a case of interhemispheric SCE detected by computed tomography (CT) after percutaneous coronary intervention.

**Patient concerns:**

A 71-year-old man suddenly lost consciousness and fainted 2 hours prior with a head trauma history. Percutaneous coronary intervention was performed on the second day.

**Diagnoses:**

Head CT findings showed that the anterior longitudinal fissure of the brain was banded with high density and was uneven in thickness. The edge of the falx side of the brain was straight, smooth, and sharp, and the edge of the brain parenchyma was clear, without obvious edema or a space-occupying effect.

**Interventions:**

Ticagrelor was given as an antiplatelet therapy; analgesic, antispasmodic symptomatic and supportive treatment was also administered.

**Outcomes:**

Two days later, the band-like high density between cerebral hemispheres was completely absorbed, and the patient's condition improved and his headache resolved.

**Lessons:**

SCE is relatively uncommon during or after the intravascular injection of contrast media. Familiarity with the clinical features and CT findings of SCE may increase clinicians’ awareness of this disease, thus avoiding potential misdiagnosis and mistreatment.

## 1 Introduction

Subdural contrast extravasation (SCE) is a rare and possible complication that can arise following the intravascular injection of a contrast agent. Its manifestations are similar to subarachnoid hemorrhage (SAH) or acute subdural hematoma (ASDH). The difference between these different conditions is of great significance for treatment.^[1,2]^ Clinicians’ overall understanding and knowledge of SCE is low, which can lead to misdiagnosis and mistreatment. Here, we report on a case of interhemispheric SCE shares similarities with interhemispheric subdural hematoma (IHSDH) following percutaneous coronary intervention. We reviewed the previous literature to explain this rare phenomenon and improve upon the available knowledge on and diagnosis of SCE. Written informed consent was obtained from the patient for the publication of this case report and any accompanying images.

## 2 Case report

A 71-year-old man suddenly lost consciousness and fainted 2 hours prior to admission, which was preceded by a history of trauma to his head. The patient experienced a headache and discomfort after waking. He had a history of hypertension, diabetes, hyperuricemia, gout, hyperlipidemia, and atherosclerosis. Blood biochemistry and blood gas examination showed hyperkalemia (K^+^5.56 mmol/L), metabolic acidosis, and abnormal renal function.

**Figure 1:**
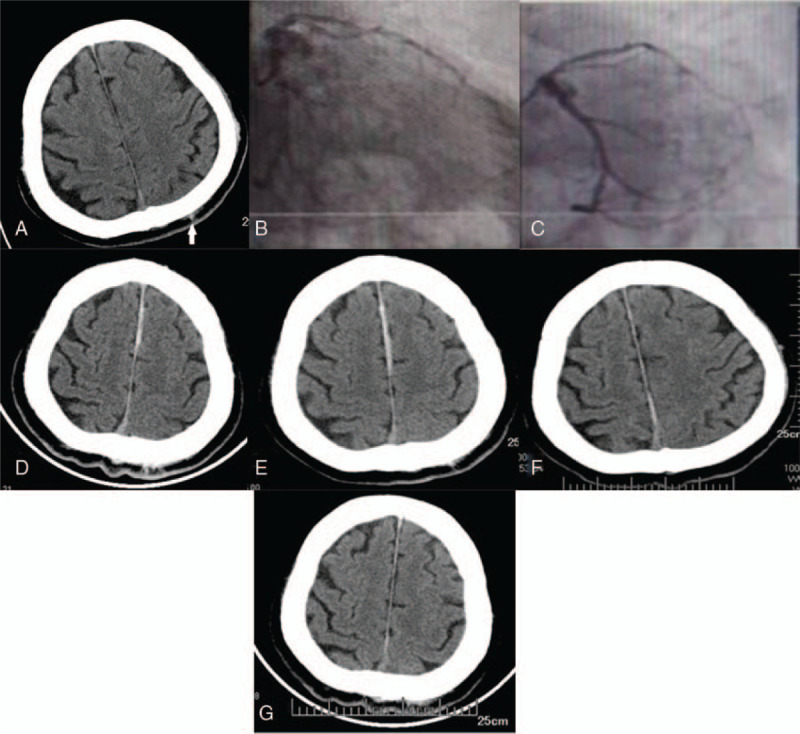
A 71-year-old man suddenly lost consciousness and fainted 2 hours prior to admission with a history of trauma to his head. The patient experienced a felt headache and discomfort upon waking. (A) Initial head CT revealed a small, left parietal subcranial hematoma (arrow). (B) Left coronary angiography finding showed that the wall of the left anterior descending artery was irregular, the proximal segment was severely stenosed, the left circumflex branch was thick, the wall was irregular, and the middle and distal segments showed moderate stenosis. (C) After percutaneous coronary intervention, left anterior descending artery stent expansion was satisfactory without residual stenosis. (D) Head CT following percutaneous coronary intervention showed that the anterior longitudinal fissure of the brain exhibited a band-like, high density with uneven thickness (about 2–5 mm), and the CT value was about 59–70 HU, with an average of 65 HU. The edge of the falx side of the brain was straight, smooth, and sharp, and the edge of the parenchymal side was clear without obvious edema or a space-occupying effect. (E) The high density range of the anterior longitudinal fissure increased at 5 hours. (F) At 22 hours, the lesions were basically absorbed. (G) Head CT re-examination suggested that the banded high density in the cerebral hemisphere was completely absorbed 2 days later.

Computed tomography (CT) of the head revealed a small, left parietal subcranial hematoma (Fig. 1A) 2 hours after symptom onset. A chest CT examination showed enlargement of the heart and calcification of the coronary artery. On the second day, coronary angiography and percutaneous coronary intervention were performed. During the operation, severe stenosis of the proximal left anterior descending coronary artery and moderate stenosis of the middle and distal left circumflex artery were found (Fig. 1B). During the operation, one drug-eluting stent was implanted in the stenosis of the left anterior descending artery. The stent expansion was satisfactory and no residual stenosis was found after repeated angiography (Fig. 1C). Symptomatic and supportive treatments including dual antiplatelet therapy, lipid-regulating, plaque-stabilizing, and antihypertensive therapy were given.

The patient felt an obvious headache after operation. Postoperative head CT re-examination (Fig. 1D) showed that the anterior longitudinal fissure of the brain exhibited a band-like high density with uneven thickness (about 2–5 mm), and the CT value was about 59–70 HU, with an average of 65 HU. The edge of the falx side of the brain was straight, smooth, and sharp, and the edge of the parenchymal side was clear without obvious edema and or a space-occupying effect. The initial diagnosis, as determined by CT, was “SCE”; however, it needed to be differentiated from ASDH and SAH. Therefore, two follow-up head CT re-examinations were conducted within 24 hours. The high-density range of the anterior longitudinal fissure increased at 5 hours (Fig. 1E). At 22 hours, the lesions were basically absorbed (Fig. 1F), so the final diagnosis was SCE. Two days later, head CT re-examination (Fig. 1G) suggested that the banded high-density in the cerebral hemisphere was completely absorbed. The patient's condition improved and his headache resolved.

## 3 Discussion

SCE is rare and refers to the leakage of contrast media into the subdural space during or after intravenous injection of contrast media. When the contrast agent appears between the 2 cerebral hemispheres, its manifestations are similar to ASDH or SAH, and the difference between these conditions is of great significance in the treatment of patients.

Contrast agent extravasation into the cerebrospinal fluid or brain parenchyma is usually asymptomatic, or it can manifest as encephalopathy, seizures, cortical blindness, and focal neurological deficits, such as ophthalmoplegia. Neurological findings generally appear 2–12 hours after contrast injection.^[3]^ In clinical settings, SCE is less specific and can be difficult to distinguish from IHSDH or SAH; as such, it is essential to exclude SCE by imaging examination.

Noncontrast CT is of great value when differentiating between SCE, IHSDH, and SAH. The radiological features of extravasated contrast medium include diffuse hyper-attenuation of the cortical parenchyma without an evident relationship to a particular vascular territory. Since the CT attenuation value of contrast agents in the blood (normally > 200 HU)^[4]^ is higher than that of hematomas (normally 60–80 HU),^[5]^ the attenuation values are higher for extravasated contrast than for blood.^[6]^ It has been reported^[2,7]^ that the CT value of extravasation contrast agent (range: 91–274 HU) exceeds that of hematoma (range: 28–92 HU). When the CT attenuation value is > 100 HU, it can be directly diagnosed contrast agent extravasation. Although the contrast agent is diluted in the cerebrospinal fluid or in cases of preexisting effusion,^[8]^ it can result in a reduction of the CT attenuation value. The CT value in this case was 65 HU, which rendered it difficult to achieve a definitive diagnosis.

Noncontrast CT shows that SAH produced anterior interhemispheric hyperdensity only, with a zigzag contour and extension from the calvarium to the rostrum of the corpus callosum. A long, thin band of increased attenuation in the region of the falx cerebri (the falx sign) has been regarded as evidence of SAH. However, IHSDH produces unilateral crescentic hyperdensities that are greatest in the posterior superior part of the fissure, located behind and above the splenium of the corpus callosum with a flat medial border and a convex lateral border. Shorter, wider, crescentic configuration or wedge-shaped interhemispheric fluid collections of blood-equivalent attenuation have been considered representative of IHSDH. Despite the density of the contrast material flooding the subarachnoid space, there is no change to the appearance of the posterior fissure combination.^[9–11]^ In this case, the hyperdensity was located in the anterior longitudinal fissure and featured a smooth edge. Therefore, SCE should be considered first, and short-term follow-up is recommended to exclude ASDH. CT examination can demonstrate whether the contrast agent in the subdural space resolves within 24 to 72 hours; the time of complete clearance of subarachnoid blood or ASDH is usually longer.^[12]^ Contrast media-induced neurotoxicity has been reported as transient and rapidly reversible.^[13]^ It usually appears soon after the administration of a contrast agent. In this case, the subdural high density in the cerebral hemisphere was found early after the percutaneous coronary intervention, and the high density disappeared completely within 24 hours. In addition, the patient had a history of percutaneous coronary intervention, so we took SCE, rather than ASDH, into consideration. The CT manifestations described herein can be used as points for identification.

The mechanism of SCE is still unclear. Many scholars^[14–16]^ believe that SCE is related to the disruption of the blood–brain barrier (BBB). When a contrast agent is injected into the artery, it causes transient osmotic destruction. Severe hypertension and atherosclerotic embolism secondary to coronary intervention can cause cerebral ischemia. When cerebral ischemic injury exists, the BBB may break down, and the intravascular contrast agent can penetrate it and leak into the subdural space. It has also been reported that the increased permeability of contrast media is caused by temporary dural vascular rupture, which is due to the neurotoxic effects of highly permeable contrast media or vascular injury caused by hypertension, ischemia, or hypoxia. As such, the contrast media can diffuse into the subdural space through the ruptured dural vessels.^[1,2,6]^ Most patients do not have SCE following coronary angiography examination. However, in this case, cerebral ischemia, the neurotoxic effects of a hyperosmolar contrast agent, and invasive nature of coronary angiography potentially led to a temporary vascular injury that affected the BBB and resulted in dural blood vessel rupture. This ultimately led to the leakage of the contrast agent into the subdural space. In addition, the patient had a prior history of a minor trauma injury of the head. Although there were no obvious signs of bleeding, the BBB is likely to be damaged. Contrast agent may enter the subdural space through the damaged BBB upon the intracoronary injection of contrast agents.

This report has several limitations. Dual-energy CT, a relatively new technology,^[17–19]^ is advantageous as it can identify the attenuation differences between 2 energies; thus it can differentiate between iodine contrast medium and hematoma, and can facilitate the detection of contrast media. However, this new technology has not been routinely carried out in our hospital. As such, we hope that we will be able to apply energy spectrum CT when encountering similar cases in the future.

In conclusion, SCE is uncommon, but the possibility of SCE should be considered in cases where there is an accumulation of subdural contrast media during or after intravascular injection of said media. Finally, it should be noted that specific imaging findings, including a smooth and clear boundary, a high CT attenuation value, and density changes on short-term follow-up can also aid in achieving an accurate diagnosis.

## Author contributions

**Validation:** Kefu Liu.

**Visualization:** Jian Huang, eryan Sheng.

**Writing – original draft:** Jinghua Chen, Ping Xie.

**Writing – review & editing:** Jinghua Chen, Kefu Liu.
